# Transcriptomic analysis of granulosa cell populations proximal and distal to the germinal disc of chicken preovulatory follicles

**DOI:** 10.1038/s41598-021-84140-w

**Published:** 2021-02-25

**Authors:** Guoqiang Zhu, Chao Fang, Chunheng Mo, Yajun Wang, Yan Huang, Juan Li

**Affiliations:** 1 Key Laboratory of Bio-Resources and Eco-Environment of Ministry of Education, College of Life Sciences, Sichuan University, Chengdu, 610065 People’s Republic of China; 2 The China Conservation and Research Center for the Giant Panda, Wolong, People’s Republic of China

**Keywords:** Transcriptomics, Reverse transcription polymerase chain reaction

## Abstract

Within the oocytes of chicken preovulatory follicles, the engulfed yolk constitutes 99% of the oocyte content, while the small germinal disc (GD) (which contains the nucleus and 99% ooplasm) occupies only less than 1%. Relative to the position of the GD, the single granulosa cell layer surrounding the oocyte can be sub-divided into two sub-populations: granulosa cells proximal (named Gp cells) and distal (Gd cells) to the GD. It was reported that Gp cells and Gd cells differ in their morphology, proliferative rate and steroidogenic capacity, however, the underlying mechanism controlling granulosa cell heterogeneity remains unclear. Here we analyzed the transcriptomes of Gd and Gp cells of preovulatory (F5 and F1) follicles in chicken ovaries. We found that: (1) genes associated with cell cycle and DNA replication (*CDK1*, *CCNB3* etc.) have comparatively higher expression levels in Gp cells than in Gd cells, while genes associated with steroidogenesis (*CYP51A1*, *DHCR24*) are highly expressed in Gd cells, indicating that Gp cells are likely more mitotic and less steroidogenic than Gd cells; (2) genes associated with extracellular matrix remodeling, cell adhesion and sperm binding (ZP3, ZP2) are differentially expressed in Gp and Gd cells; (3) Furthermore, signaling molecules (WNT4/IHH) and receptors for NGF (NGFR), epidermal growth factor (EGFR), gonadotropins (FSHR/LHR) and prostaglandin (PTGER3) are abundantly but differentially expressed in Gp and Gd cells. Taken together, our data strongly supports the notion that Gp and Gd cells of preovulatory follicles differ in their proliferation rate, steroidogenic activity, ECM organization and sperm binding capacity, which are likely controlled by gonadotropins and local ovarian factors, such as GD-derived factors.

## Introduction

Similar to those in other vertebrates, the ovary in adult birds contains numerous developing follicles in different size. Under the control of pituitary hormones [follicle-stimulating hormone (FSH) and luteinizing hormone (LH)] and local ovarian factors, such as somatic cell-derived factors [epidermal growth factors, activins/inhibins, kit ligands (KL), bone morphogenic proteins (BMPs)) and oocyte-derived growth factors [growth differentiation factor 9 (GDF9) and bone morphogenic protein (BMP15)), these follicles undergo –growth, selection, maturation and finally are released into the oviducts for fertilization^1–3^.

It is clear that in vertebrates, such as in mammals, each ovarian follicle has a complex structure. The oocyte is encircled by granulosa cells (GC) and theca cells. The oocyte growth depends on these cells for nutrients transportation and mechanical support. Theca cells do not undergo obvious change in their morphology and function during follicular development. In the early stage, GC undergo dramatic morphological change, transiting from a squamous cell shape into a cuboidal one^1^. In the later stages (eg antral follicles and preovulatory follicles), GC can be further divided into mural GC which are distal to the oocyte and cumulus GC which surround the oocyte. GC seems to play key roles in orchestrating oocyte-somatic cell crosstalk and the secretion of steroids and peptide hormones (e.g. progesterone, and inhibins)^4,5^, thus coordinating oocyte development and the release of pituitary gonadotropins (eg FSH/LH).

Chicken ovaries also contain numerous developing follicles in different size, including cortical follicles (< 1 mm in diameter), white follicles (1–5 mm), yellow follicles (5–8 mm), and five to six large preovulatory follicles (10–40 mm) which are under a clear hierarchy in ovulation. The largest preovulatory follicle (named F1 follicle) is ovulated first, while the second to fifth/sixth preovulatory follicles (F2 to F5/F6) will be ovulated sequentially at an interval of 24–26 h^6^. Owing to the large amount of lipid-rich yellow yolk accumulation in these preovulatory follicles, the nuclei and 99% of the ooplasm concentrate in a small region of 1–2 mm in diameter, thus is designated as the germinal disc (GD), which can be observed as a small white plaque at the surface of the large oocyte under the microscope^7,8^. Relative to the position of the GD, GC encircling the oocytes can be sub-divided into two subpopulations: Gp cells which are located proximal to the GD, and Gd cells which are distal to the GD^7,9^.

Electron microscopy analyses revealed that Gp cells might vary in their width, while the shape of Gd cells was mostly uniform^7–9^. In addition, Gp cells have a greater percentage of cells in mitotic stage with columnar shape, while Gd cells display cuboidal shape^7–9^. In addition to their morphological difference, Gd cells and Gp cells differ in their total protein content and mitotic activity. Gp cells showed lower protein content and higher mitotic activity, with a relatively higher proportion of cells in S and G2/M phase, whereas Gd cells demonstrated a higher protein content and a higher proportion of cells in G1 phase, as detected by flow cytometry^10^. In the largest F1 preovulatory follicle, Gp cells show a higher ^3^H-thymidine incorporation rate^11,12^, however, Gd cells show a greater responsiveness to LH and produce more progesterone^13^, which is likely associated with the higher mRNA levels of LH receptor therein^14^.

Although the two subpopulations have been reported to differ in their morphological features, mitotic activity and steroidogenesis, the molecular mechanism behind these differences remain unclear. In the present study, Gp cells and Gd cells proximal and distal to the GD were isolated from chicken F5 and F1 preovulatory follicles and subjected to transcriptome analyses. Results from our study, for the first time, provided global gene expression profiles of the two sub-populations of GC. This study will not only help to address their morphological and functional differences, but also provide valuable clues on how oocytes (or the GD) and pituitary hormones coordinate the morphological and functional diversity observed in GC, a phenomenon which has also been observed in mammalian preovulatory follicles.

## Materials and methods

### Animal tissues and sub-population of granulosa cells separation

**Figure 1:**
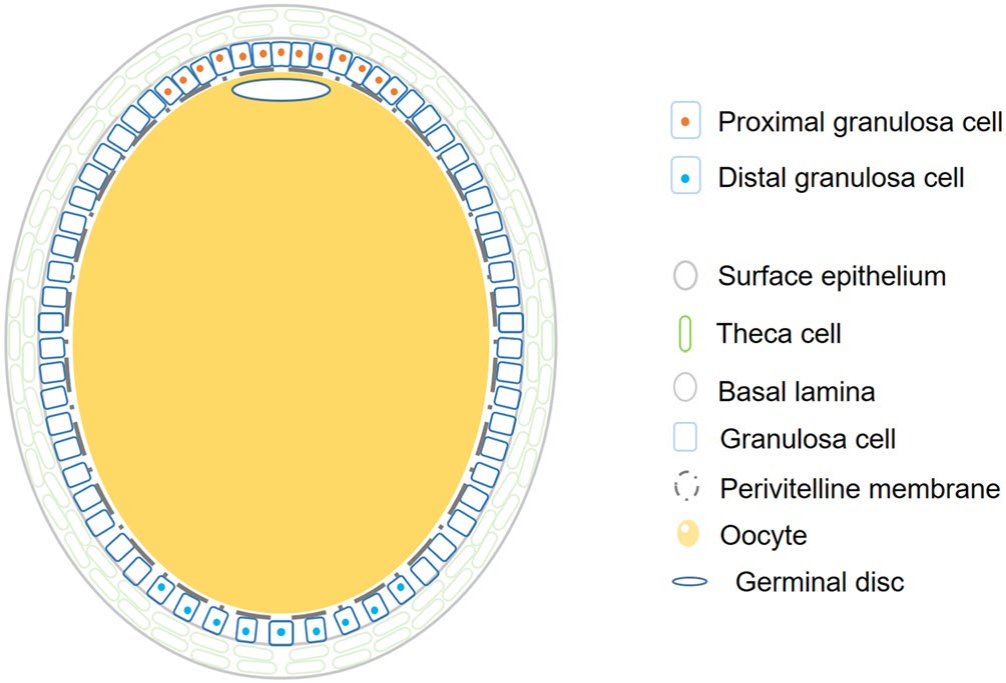
The structural model of chicken preovulatory follicle. The granulosa cells above the germinal disc (Gp) and the granulosa cells distal to the germinal disc (Gd) are shown.

Egg-laying hens with normal follicular hierarchies (Lohmann Layer strain) were kindly provided by MUXING company in Sichuan, China. In this study, a total of 15 hens in the same peak laying period were subjected for sample collection. Five were sampled for the transcriptome analyses, the remaining 10 birds were sampled for real-time PCR. The F5 follicle and F1 follicle were collected from each bird. In general, a chicken ovary in peak laying period contains 30–100 small yolky follicles and 4–7 follicles recruited in the hierarchy. F5 and F1 follicles selected in this study belonged to the third growth phase with fast growth in size and rapid yellow yolk incorporation^6^. The F5 follicle and F1 follicle were collected from each bird. Granulosa cells were then separated from neighboring cells. Following our previously established method^5,15^, ~ 8 mm sections of granulosa cells proximal and distal to the GD (visually identified as a white plaque) were isolated for RNA extraction. A schematic illustration of the cell localization in the follicle is shown in Fig. 1.

### RNA extraction, RNA-seq library construction and sequencing

According to the manufacturer’s instructions and our established protocol, total RNA was extracted and then dissolved in diethylpyrocarbonate-treated H_2_O (DEPC-H_2_O) for quality and quantity evaluation. Briefly, the isolated granulosa cell subpopulations were dispersed in RNAzol reagent (500 μL) (Molecular Research Center, Cincinnati, OH, USA) for lysis through a pipette several times and then shaken vigorously 15 s and stored for 5–10 min at room temperature (4–25 °C). The extract was then centrifuged at 12,000*g* for 15 min at room temperature. The supernatant was transferred to a new tube with 4-bromoanisole added (0.5% of the supernatant volume) (MRC, BN191). The resulting mixture was shaken and then subjected for centrifugation at 12,000*g* for 10 min at room temperature. After centrifugation, the RNA-containing supernatant was then transferred to a new tube for precipitation with equal volume of isopropanol added (v/v). The mixtures were stored at room temperature for 10 min and centrifuged at 12,000*g* for 10 min. The RNA pellet was washed with 75% ethanol and air dried before solubilization in 10 μL DEPC-H_2_O and stored in − 80 °C freezer before further analyses.

RNA-seq libraries were prepared following the standard Illumina protocols by Novogene (Beijing, China). In brief, mRNA (at least 3 μg, equally pooled from 5 hens) were enriched from total RNA by poly-A oligo-attached magnetic beads with an integrity value > 8.0. Double-stranded complementary DNA was synthesized with random hexamer primers and purified with AMPure XP beads. Inserts with expected size were concentrated and subjected for transcriptome analyses.

### Differential gene expression analyses

In the current study, transcriptomic alignments were performed using Tophat v2.0.12 against the reference genome of *Gallus gallus* (ftp://ftp.ensembl.org/pub/release-72/fasta/gallus_gallus/dna)^16^. Transcript counts were estimated using HTSeq v0.6.1 with default parameters^17^. Based on the sequencing depth and gene length of the read count, FPKM (Fragment reads Per Kilobase per Million mapped reads) was chosen in this study as the key parameter in gene expression analyses. Differential gene expression was analyzed using count data from the TopHat‐HTSeq pipeline was by DESeq v1.42.0^18^. A q-value of < 0.05, which was adjusted from p-value by the Benjamini&Hochberg method, was employed in this study and the 1.5-fold minimum differential expression was designated as the threshold of differentially expressed genes^19^.

### Functional gene annotation

Kyoto Encyclopedia of Genes and Genomes (KEGG) enrichment analyses and Gene ontology (GO) analyses were performed by GOSeq Release2.12 and KOBAS v2.0 respectively^20,21^. The terms with q-value < 0.05 were considered significantly enriched. The online database resource search tool for the retrieval of interacting Genes/Proteins (STRING, http://string-db.org/) was employed for protein–protein interaction analyses. String score higher than 700 was collected and then subjected to further analyses by Cytoscape v3.3.0 software^22^.

### Reverse transcription and quantitative real-time PCR validation

For reverse transcription, oligodeoxythymide (0.5 μg) and total RNA (2 μg) were mixed in a total volume of 5 μL, and incubated at 70 °C for 10 min, then cooled at 4 °C for 2 min. 1 μL single strand buffer, 0.5 μL each deoxynucleotide triphosphate, 0.5 μg oligo-deoxythymidine, and 100U moloney murine leukemia virus (MMLV) reverse transcriptase (Promega, Madison, WI, USA) were then added to the reaction mix in a total volume of 10μL. Reverse transcription (RT) was performed at 42 °C for 90 min. The total reaction mix containing the first-strand cDNA was diluted ten times by the addition of 90 μL Milli-Q water and stored − 20 °C freezer for downstream application.

Quantitative real-time PCR was employed in this study to validate the mRNA level of selected genes. Primers used for the amplification of these target genes were listed in Table 1. According to our previously established method^23,24^, real-time PCR was conducted on CFX96 real-time PCR Detection System (Bio-Rad, Hercules, CA, USA) in a total volume of 20 μL with 13.2 μL Milli-Q water, 2 μL RT product, 1 μL single PCR buffer, 0.5 μL DMSO, 0.4 μL 2 mM each dNTP, 0.3 μL 20 μM primer, 0.3 μL Taq DNA polymerase (Invitrogen, Carlsbad, CA, USA), and 1 μL EvaGreen (Biotium Inc., Hayward, CA, USA). The specificity of PCR amplification was first checked by agarose gel electrophoresis and then subjected to sequencing for checking the identity of all PCR products. The amplification condition of real-time PCR was as follows: pre-denaturation at 94 °C for 2 min, then with 40 cycles of denaturation at 94 °C for 20 s, annealing at 60 °C for 15 s, and extension at 72 °C for 20 s. The mRNA level of these target genes was first normalized as the ratio to the housekeeping genes *GAPDH*, and then expressed as the fold difference to the control group. The qPCR data of the four experimental groups (n = 10 each) was analyzed by the comparative Ct method^25^, and tested for normality using Student’s *t* test or by one-way ANOVA followed by the Dunnett’s test using GraphPad Prism 6.01 (GraphPad Software, San Diego, CA). To validate the results, all quantitative real-time PCR experiments were repeated at least twice.

**Table 1:** Primers used for quantitative real-time PCR validation.

Gene name	Sense/antisense	Primer sequences	GeneBank accession
*CDC20*	Sense	AAGCCACAGAATGCTCCAGAAGG	NM_001006536.1
	Antisense	CAGAGCCACTGCCAGGAAGTTC	
*CCNB2*	Sense	GGAGAATGCCGTGACTGGACATAA	XM_015278893.2
	Antisense	CCTTTCGTAGCCTTTACTGGTGGTT	
*CCNA1*	Sense	GGAACGGCAGCAATTCTTCTGG	XM_015278427.2
	Antisense	ACTGGTTGATGGTTGGCACTGT	
*RFC4*	Sense	CCAGCAACAGAGGCTATTGGATGT	NM_001006550.2
	Antisense	CAGTGACAGTCTTCTCCGTGATCTC	
*MCM2*	Sense	GCTACAGAGTTCACGATCCAGAGG	NM_001006139.1
	Antisense	CTTCAGCAGCTCAGCAGTTCCT	
*CYP51A1*	Sense	GGTTATCCAGAAGCGTCGGAGTT	NM_001048077.1
	Antisense	CAGGAGCAGCCCAATGAGCAT	
*DHCR24*	Sense	GCTGACCGCATACCTGAATCCTAT	NM_001031288.1
	Antisense	GAACATCTCACGAGGCTTCCATCA	
*LHCGR*	Sense	TCAGGCGGATACACAACGATGC	NM_204936.1
	Antisense	AGGCGGCAGTCTCTTCAGTG	
*FSHR*	Sense	GCACCTTCCAAGCCTCAGAT	NM_205079.1
	Antisense	AGAACTCAGGCCCATGAACG	
*EGFR*	Sense	GGACTTCGAGAGCTGCCAATGA	NM_205497.2
	Antisense	TGTGAGAGGCTTCCTGCTTGTATC	
*NGFR*	Sense	CTCCGATGCTGAGTGCAGAGAC	NM_001146133.1
	Antisense	GCCCATGACGGTGGTGACAA	
*PTGFR3*	Sense	CTTGCATTTGCTGGAGTCCATTCC	NM_001040468.1
	Antisense	TCTGATTCCATGTTGCCATCCG	

### Ethics approval

In the present study, the handling of animals followed the animal welfare recommendations and were approved by the Animal Ethics Committee of Sichuan University. All the experiments were performed according to the regulations and guidelines established by the Ministry of Science and Technology of the People’s Republic of China (Approval number: 2006-398).

## Results

### Transcriptomic analyses of Gp and Gd granulosa cells in F5 and F1 preovulatory follicles

In this study, Gp and Gd cells were collected from F5 and F1 follicles, and subjected to high-throughput RNA sequencing analyses. The sequencing data was deposited in the NCBI public database (accession number: GSE118886).

As shown in Fig. S1, a total of 104 genes were found to be differentially expressed in granulosa cell proximal to the GD (G5p cells) and distal to the GD (G5d cells) of F5 follicles. Among them, 78 genes showed a higher abundance in G5p cells and 26 genes showed a higher abundance in G5d cells. The top 25 up-regulated genes and down-regulated genes were listed in Table 2.

**Table 2:** Top 25 genes showing significant up or down regulation tendency in granulosa cells proximal and distal to the germinal disc (GD) in F5 follicles (q < 0.05).

Gene name/Gene_ID	Fold change (Log 2 )	Gene name/Gene_ID	Fold change (Log 2 )	Gene name/Gene_ID	Fold change (Log 2 )
**Higher abundance in F5 proximal versus distal granulosa cells**					
*BMP15*	5.34	*FAM101B*	3.53	ENSGALG00000023172	2.93
Novel00730	5.17	*NGFR*	3.45	*MRC2*	2.91
*ENG*	4.59	*CLDN1*	3.43	*IRF6*	2.81
*TMEM184A*	4.59	*B3GNT7*	3.34	*PLK3*	2.80
*ABCC4*	3.99	*EMCN*	3.34	ENSGALG00000007703	2.48
*AMH*	3.68	*ZP2*	3.34	*C6orf132*	2.41
*PPP1R3B*	3.66	*ADAMTSL3*	3.31	*RBPMS2*	2.40
*CHRDL1*	3.62	*CCNO*	3.25		
*WT1*	3.53	*NBL1*	3.04		
**Lower abundance in F5 proximal versus distal granulosa cells**					
*FMOD*	1.74	*LTBP2*	0.79	*RPS15A*	0.63
*STC1*	1.11	*CYP11A1*	0.70	*RPS7*	0.63
Novel01498	0.95	*FIBIN*	0.68	*RETSAT*	0.62
Novel00960	0.94	*PLTP*	0.67	*RPS6*	0.61
*FAR-1*	0.83	*RGL1*	0.67	*FNDC1*	0.61
*ADA*	0.82	ENSGALG00000028992	0.65	*RPL4*	0.60
*TGFBI*	0.81	*RPL7*	0.64	*TPST1*	0.59
*LRP1*	0.80	*PURH*	0.64		
*CTHRC1*	0.79	*SLC25A6*	0.64		

These differentially expressed transcripts were subject to Gene Ontology (GO) analyses, and found to be enriched in the biological processes associated with the single-organism developmental system, anatomical structure developmental system, developmental process, multicellular organismal development, etc.. In addition, numerous enriched differentially expressed genes were found to be related to multiple cellular components and involved in molecular functions including extracellular matrix, cell junction and extracellular region (data not shown). However, no statistical difference among the GO terms was detected between the G5p and G5d cells.

The transcriptomes of Gp (G1p) and Gd (G1d) cells in F1 follicles were also analyzed in this study. As shown in Fig. S1, 249 genes were found to be differentially expressed between G1p and G1d cells. Among them, 185 genes were found to show relatively higher expression levels in G1p cells, while 64 genes showed relatively higher mRNA levels in G1d cells. The top 50 up-regulated and down-regulated genes were listed in Table 3.

**Table 3:** Top 50 genes showing significant up or down regulation tendency in granulosa cells proximal and distal to the germinal disc (GD) in F1 follicles (q < 0.05).

Gene name/Gene_ID	Fold change (Log 2 )	Gene name/Gene_ID	Fold change (Log 2 )	Gene name/Gene_ID	Fold change (Log 2 )
**Higher abundance in granulosa cells proximal (G1p) versus distal (G1d)**					
Novel00730	7.42	*CCNO*	5.22	*CA9*	4.34
*SIDT1*	6.96	*BARD1*	5.12	*NUSAP1*	4.31
ENSGALG00000007703	6.61	*CORO2B*	5.11	*RGS16*	4.26
*IRF6*	6.33	*ZP2*	5.03	*HES1*	4.26
ENSGALG00000026301	6.31	*EPCAM*	5.01	ENSGALG00000023172	4.19
*AKR1D1*	6.17	*Smad7a*	4.96	*TMEM184A*	4.18
*MRC2*	6.16	*TMEM72*	4.95	*TRAIP*	4.13
*PPP1R3B*	6.16	*EMCN*	4.75	*SVEP1*	4.06
*BRCA1*	6.07	*DGAT2*	4.73	*NBL1*	4.00
*NGFR*	5.93	*Wnt6*	4.67	*SNX22*	4.00
*GTSE1*	5.92	*NUF2*	4.56	*SGK2*	3.92
*ENG*	5.82	*WNT4*	4.53	*CCNB2*	3.84
*RRM2*	5.75	*SH3RF3*	4.51	*MCAM*	3.77
*CLDN1*	5.73	*FAM101B*	4.50	*KIF23*	3.76
*WT1*	5.62	*CDCA7*	4.49	*CDK1*	3.69
*INHBB*	5.45	*AURKA*	4.48	*CDH3*	3.66
*WEE2*	5.39	*KIF2C*	4.35		
**Lower abundance in granulosa cells proximal (G1p) versus distal (G1d)**					
ENSGALG00000028371	2.15	*KIAA0040*	0.95	*LSS*	0.75
ENSGALG00000009777	1.62	*SMTN*	0.94	*SLC5A6*	0.73
*TNR*	1.58	*MVD*	0.94	*TCN2*	0.73
*FMOD*	1.36	*fn1*	0.93	*FAR-1*	0.73
*IGSF10*	1.34	*CSRP1*	0.93	*DHCR7*	0.72
Novel00960	1.33	*PLTP*	0.90	ENSGALG00000026545	0.72
*SLC45A3*	1.32	*MMD*	0.90	*RETSAT*	0.71
*FADS2*	1.27	*STAR*	0.86	*ACAT2*	0.70
*CYP11A1*	1.21	*IGFBP2*	0.86	*LDHB*	0.69
*STRA6*	1.20	*ALAS1*	0.85	*ZP3*	0.67
*ALB*	1.19	Novel01841	0.82	*BRT-1*	0.66
*KIAA1324*	1.14	Novel00919	0.81	*SLC25A6*	0.63
*STC1*	1.10	*NPC2*	0.80	*FNDC1*	0.63
ENSGALG00000019312	1.08	*TGFBI*	0.80	*SQLE*	0.63
*RIL*	1.04	*DKK3*	0.78	*PGRMC1*	0.63
ENSGALG00000027090	1.01	*SC5D*	0.76	*PEBP1*	0.63
*ACLY*	0.99	*ADAMTS3*	0.76		

**Figure 2:**
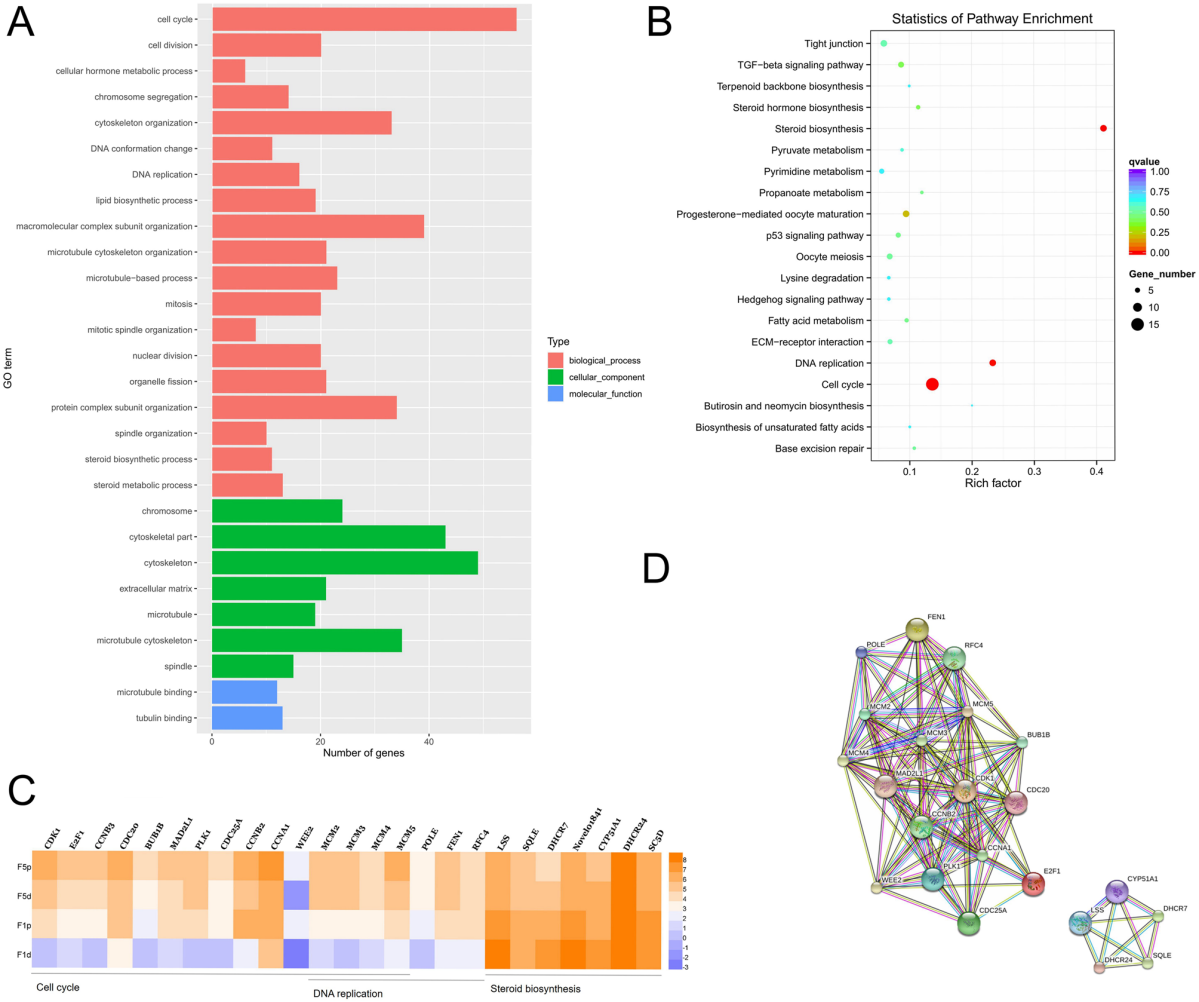
The differential gene expression profile of the granulosa cells (Gp and Gd) in F1 follicles. (**A**) Gene ontology (GO) functional enrichment of genes differentially expressed in the granulosa cells from F1 follicles. The y-axis and x-axis indicate the number of genes in each cluster and the names of clusters respectively. (**B**) Scatter plot of enriched KEGG pathways for differentially expressed genes in Gp and Gd granulosa cells from F1 follicles. The rich factor is the ratio of the differentially expressed gene number to the total gene number in a certain pathway. The size and color of the dots represent the gene number and the range of the q-value, respectively. (**C**) The heatmap displaying the genes enriched in cell cycle, DNA replication and steroid synthesis in KEGG analysis. The red colour represents the transcript over-expression or up-regulated while the yellow colour represents the transcript down-regulated. (**D**) The cell cycle, DNA replication and the steroid synthesis network identified from G1p and G1d granulosa cells based on STRING database.

The 249 differentially expressed genes in G1p and G1d cells were then subjected to GO analyses (Fig. 2A). They were significantly enriched in biological processes, cellular components and molecular functions. 67 GO terms were categorized into different biological processes. For instance, 20 genes associated with mitosis (GO: 0007067), 20 genes associated with cell division (GO: 0051301), 11 genes associated with steroid biosynthesis (GO: 0006694), 16 genes associated with DNA replication (GO: 0006260), 17 genes associated with cell cycle phase transition (GO: 0044770) and 19 genes associated with lipid biosynthetic process (GO: 0008610). In addition, 16 GO terms identified fell under the category of cellular components including 19 genes associated with microtubule (GO: 0005874), 21 genes associated with extracellular matrix (GO: 0031012), 79 genes associated with extracellular region (GO: 0005576) and 49 genes associated with cytoskeleton (GO: 0005856). 7 GO terms identified were related to molecular function including 12 genes associated with microtubule binding (GO: 0008017) and 13 genes associated with tubulin binding (GO: 0015631). Statistical differences were observed in each of the above GO terms identified between G1p and G1d cells.

Kyoto Encyclopedia of Genes and Genomes (KEGG) pathway analysis was performed in G1p and the G1d cells. As shown in Fig. 2B, with the threshold of q-value < 0.05, the differentially expressed genes observed in G1p and G1d cells from F1 follicles concentrate in the three pathways, including 15 genes associated with cell cycle (gga04110), 7 genes associated with DNA replication (gga03030) and 7 genes associated with steroid biosynthesis (gga00100) (Fig. 2B).

To illustrate the gene expression profile of Gd and Gp cells in the two follicular stages, we depicted a heatmap of genes involved in top 3 GO terms, namely cell cycle, DNA replication and steroid synthesis. As shown in Fig. 2C, the genes associated with cell cycle (*CDK1*, *E2F1*, *CCNB3*, *CDC20*, *BUB1B*, *MAD2L1*, *PLK1*, *CDC25A*, *CCNB2*, *CCNA1*, *Wee2*, *MCM2*, *MCM3*, *MCM4* and *MCM5*) showed relatively higher expression levels in G1p cells than in G1d cells. Moreover, the genes involved in the DNA replication (*MCM2*, *MCM3*, *MCM4*, *MCM5*, *POLE*, *FEN1* and *RFC4*) also showed relatively higher mRNA levels in G1p than in G1d cells. In contrast, the genes involved in steroid synthesis (*LSS*, *SQLE*, *DHCR7*, *Novel01841*, *CYP51a1*, *DHCR24*, *SC5D*) showed relatively higher expression levels in G1d cells than in G1p cells.

To investigate on how these genes interact, protein–protein interaction analyses based on the STRING database was performed. It showed that a total of 17 genes (*FEN1*,* POLE*,* RFC4*,* MCM2*,* MCM3*,* MCM4*,* MCM5*,* MAD2L1*,* CDK1*,* BUB1B*,* CCNB2*,* CDC20*,* WEE2*,* PLK1*,* CCNA1*,* E2F1* and *CDC25A*) integrated the network involving DNA synthesis and replication, while 5 genes (*CYP51A1*,* LSS*,* DHCR7*,* DHCR24* and *SQLE*) integrated into the steroid synthesis network (Fig. 2D).

### Validation of the differential gene expression in F5 and F1 preovulatory follicles

**Figure 3:**
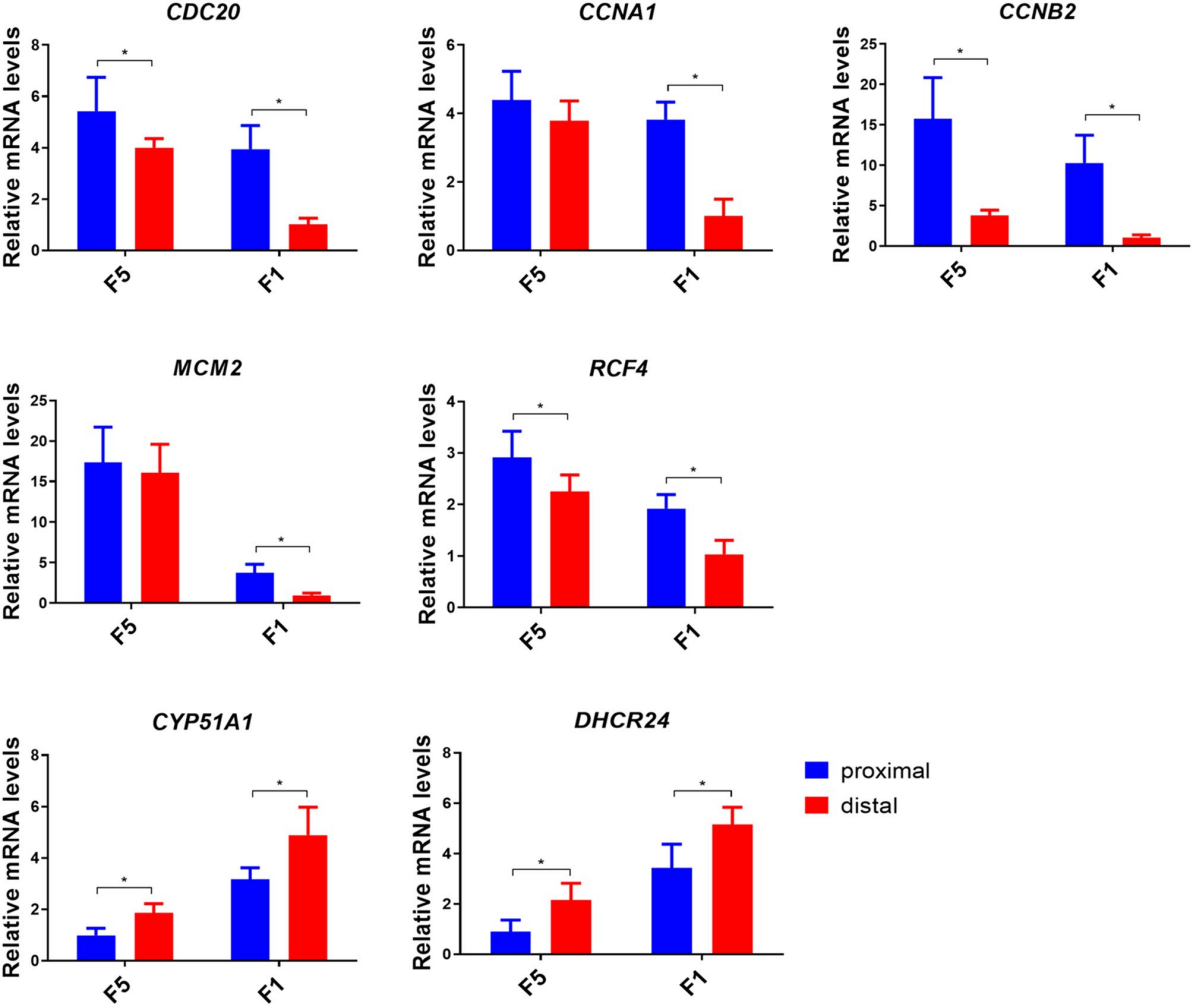
The quantitative real-time PCR validation of the genes involving cell cycle, DNA replication, steroid synthesis. The results were normalized based on the housekeeping gene *GAPDH*. Each data point represents mean of ten individuals (N = 10) *p < 0.05.

To validate the differential expression patterns of genes identified by RNA-seq analyses, quantitative RT-PCR (qRT-PCR) was employed in this study. As shown in Fig. 3, the differential expression patterns of genes involved in cell cycle (*CDC20*, *CCNB2*, *CCNA1*), DNA replication (*RFC4*, *MCM2*), and steroidogenesis (*CYP51A1*, *DHCR24*) were confirmed to be differentially expressed between Gd and Gp cells in F1 or F5 follicles by qRT-PCR assay. These findings confirmed the reliability of RNA-seq data (Fig. 3).

In this study, genes shown as differentially expressed between F5 and F1 follicles by RNA-seq analyses were also subjected to quantitative RT-PCR (qRT-PCR) analyses. In F5 follicles, the genes include *RPL4*, *RPS6*, *CYP11A1*, *NGFR*, *PTGER3*, *WNT6*, *AMH*, *NBL1*, *FMOD*, *Id1*. In F1 follicles, the genes include *CYP11a1*, *STAR*, *NPC2*, *NGFR*, *PTGER3*, *FSHR*, *WNT4*, *WNT6*, *AMH*, *ZP2*, *ZP3*, *NBL1*, *FOMD*, *Id1*. As shown in Fig S2, these genes showed similar differential expression profiles between the two assays, thus supporting the reliability of the results.

## Discussion

It has long been proposed that in avian ovaries, granulosa cells encircling the oocyte in large preovulatory follicles show heterogeneity and can be roughly subdivided into two cell subpopulations: Gp cells which is proximal to the GD and Gd cells which is distal to the GD. These two granulosa cell subpopulations are likely to differ in their morphology, proliferative potential, and steroidogenic capacity. However, this hypothesis still lacks molecular evidence for support. In this study, we analyzed the transcriptomes of Gp and Gd cells and provided clear evidence for their heterogeneity and possible functional difference.

### Differentially expressed genes in Gp and Gd cells of large preovulatory follicles

In this study, we identified 104 genes differentially expressed in Gp and Gd cells from F5 follicles (designated as “G5p” and “G5d” cells), and 249 genes differentially expressed in Gp and Gd cells in the largest F1 preovulatory follicle (designated as “G1p” and “G1d” cells). This finding suggests a potential tendency of a higher differential expression in genes in Gp and Gd cells in follicles at a later developmental stage. This hypothesis was further supported by the GO analyses. In F5 follicles, G5p and G5d showed no statistical difference among the GO terms identified, whereas in F1 follicles, G1p and G1d subpopulations differ in each GO term with statistical significance. KEGG analysis further revealed that the genes with relatively higher expression levels in G1p cells are enriched in cell cycle and DNA replication, while the genes with higher mRNA levels in G1d cells are enriched in steroid biosynthesis.

In F1 follicles, many genes associated with cell cycle progression, e.g. *CDK1*, *E2F1*, *CCNB3*, C*DC20*, *BUB1B*, *MAD2L1*, *PLK1*, *CDC25A*, *CCNB2*, *CCNA1*, *WEE2*, *MCM2*, *MCM3*, *MCM4*, *MCM5*, etc. in **G1p cells showed higher expression levels, than in G1d cells. Among these genes, *CDK1* can function as a serine/threonine kinase and is a key player in cell cycle progression^26^. *CDC25A* activates cyclin-dependent kinases (CDKs) by removing phosphate from residues in the Cdk active site^27^. Together with Wee1, CDK1 activation is like a switch controlling the transitions from G1 to S phase and G2 to M phase. *PLK1*, the serine/threonine-protein kinase, functions as an early trigger for G2-to-M phase transition^28^. The transcription factor *E2F1* plays a key role in cell cycle transition^29^. The higher expression levels of these genes in G1p cells strongly support the idea that G1p cells have higher proliferative potentials than G1d cells, as proposed in previous studies^11,30^.

In our study, *CCNB2*,* CCNA1*,* CDC20*,* BUB1B*, and *MAD2L1* genes are also found to be differentially expressed in G1d and G1p cells. CCNB2 is essential for cell cycle control at the G2/M (mitosis) transition^31^. Cyclin A1 (*CCNA1*) may not only be involved in G2/M transition, but also be required for nuclear envelope breakdown^32,33^. Both genes showed higher expression levels in G1p cells. This finding was further confirmed by qPCR assay in our study (Fig. 3). CDC20 (cell-division cycle protein 20), as the essential regulator to activate the anaphase promoting complex (APC/C), plays an important role in cell cycle progression^34^. The gene *BUB1B* encodes a kinase, which inhibits the APC/C in kinetochore to delay the onset of anaphase and ensure proper chromosome segregation^35^. The MAD2 protein, encoded by the gene *MAD2L1*, binds to the mitotic spindle assembly to prevent the onset of anaphase until all the chromosomes are properly aligned at the metaphase plate^36^. Since all these genes showed higher expression levels in Gp cells at both follicular stages, these findings further support that G1p cells contain a greater percentage of mitotic cells.

Besides the genes associated with cell cycle, numerous genes associated with DNA replication, such as *MCM2*, *MCM3*, *MCM4*, *MCM5*, *POLE*, *FEN1*, *RFC4*, were also found to be differentially expressed between Gp and Gd cells. MCM2 and MCM7 load as a double hexamer to encircle double-stranded DNA at the origin of replication during G1 phase^37^, and they form a part of the replicative CMG helicase complex (comprising CDC45, MCM2-7 and GINS) which is essential in the final ligation step of cell cycle^38^. *POLE*, which functions as the catalytic subunit of DNA polymerase involved in DNA replication and repair^39^. FEN1, which removes the 5′overhanging flaps in DNA repair, processes the 5′ ends of Okazaki fragments in lagging strand DNA synthesis^40^. RFC4 is found to be stimulated by PCNA and forms a core complex involved in DNA-dependent ATPase activities^41^. Higher expression levels of these genes associated with DNA replication in Gp cells, also suggest that G1p cells undergo more active DNA replication than G1d cells in the largest F1 follicle. Our findings are in accordance with previous studies showing a higher ^3^H-thymidine incorporation rate in Gp cells^11,30^.

Unlike G1p cells, G1d cells abundantly express many genes associated with steroidogenesis, such as *CYP51A1*, *DHCR24*, *LSS*, *SQLE*, *DHCR7*, *Novel01841*, and *SC5D*. CYP51A1 participates in the synthesis of cholesterol by catalyzing the removal of the 14 alpha-methyl group from lanosterol^42–44^. DHCR24 regulates cholesterol homeostasis through catalyzing the reduction of the delta-24 double bond of sterol intermediates during cholesterol biosynthesis^45^. Lanosterol synthase (*LSS*) is the key four-ringed intermediate in cholesterol biosynthesis, which can convert the (S)-2,3-oxidosqualene to a protosterol cation and subsequently to lanosterol^46^. DHCR7 is the enzyme that removes the c (7–8) double bond in the B ring of sterols and catalyzes the conversion of 7-dehydrocholesterol to cholesterol. All these genes showed a higher expression level in Gd cells than in Gp cells, strongly supporting the existing hypothesis that Gd cells have a higher capacity in steroidogenesis such as progesterone synthesis than Gp cells^12,13,30^.

### Genes differentially expressed in Gp and Gd cells are associated with cell adhesion, ECM organization and sperm binding

Our transcriptome analyses revealed that many transmembrane proteins are differentially expressed in the two granulosa cell subpopulations. For example, Claudin 1 (*CLDN1*) is stably expressed in Gp cells of F5 and F1 follicles (G5p:18.49, G1p: 18.76), whereas its level is much lower in Gd cells of F1 follicles (G5d:1.61, G1d:0.3). CLDN1 are small (20–27 kDa) transmembrane proteins. As one of the most important components of tight junctions, it may form a part of the paracellular barrier to control the flow of molecules in the intercellular space between epithelium cells^47^. The high abundance of this gene in Gp cells supports the notion put forward by past studies that the existence of tight junction between Gp cells may help to prevent the incorporation of yellow yolk^8,9^. In our study, another transmembrane protein, epithelial cell adhesion molecule (*EpCAM*), which is a transmembrane glycoprotein mediating Ca^2+^-independent homotypic cell–cell adhesion in epithelial cells^48^, showed a higher abundance in Gp cells (G5p:29.02, G1p: 21.74) (G5d:7.54, G1d:0.56). EpCAM is involved in cell signaling, migration, proliferation and differentiation^49,50^. In addition, EpCAM shows the oncogenic potential via its capacity to upregulate c-myc, e-fabp, and cyclins A & E^49^. In line with the expression profile of the genes *CLDN1* and *EPCAM*, ADAMTS family members (*ADAMTS9*,* ADAMTS15*,* ADAMTSL3*) also showed similar differential expression profiles with a higher abundance in Gp cells. ADAMTS family members were reported to play important roles in connective tissue organization, coagulation and extracellular matrix remodeling^51^. In our study, ADAMTS family members 3 (*ADAMTS3*) showed the differential expression with a higher expression level in Gd cells. *TGFBI* encodes an RGD-containing protein that binds to collagens which helps to modulate cell adhesion and serve as a ligand recognition sequence for several integrins^52^, also showed a higher expression abundance in Gd cells. The gene encoding fibromodulin (*FMOD*), which participates in the assembly of the collagen fibers of the extracellular matrix and extracellular organization^53^, also showed differential expression in Gd and Gp cells. *CD151*, as a cell surface glycoprotein which collaborate with integrins to regulate cell development, activation, growth and motility, showed a higher expression abundance in Gd cells^54^. The differential expression of these genes in Gd and Gp cells strongly suggests that cell–cell junctions and extracellular matrix organization vary between Gd and Gp cells.

In this study, we also found that genes encoding zona pellucida (ZP) proteins are differentially expressed in Gp and Gd cells. The ZPs are glycoproteins and are deposited in the inner perivitelline membrane layer (PVL) between granulosa cells and oocyte, forming a three-dimensional network which play roles in oocyte protection and sperm recognition and binding^55^. As shown in Table 3, ZP3 showed a high abundance in the 4 groups (F5p: 14,379.31, F5d: 14,816.1, F1p: 82,661.9, F1d: 111,496.28). The high abundance of ZP3 concentrated in G1d cells concurs with a past report that lower protein content was detected in Gp cells by flow cytometry^10^. As the most abundant protein in PVL, ZP3 may provide the structural support during ovulation^56^. In our study, ZP3 expression differs significantly between G1p and G1d cells. This finding suggests that ZP3 may contribute to PVL architectural diversification. In mammals, ZP3 is synthesized by oocytes and suggested to bind to sperm head in O-glycan-dependent manner, thus implicated in sperm recognition^57^. Unlike mammalian oocytes, chicken oocytes are huge and their surface area may reach 35 cm^2^. Since the chicken germinal vesicle (GV) and 99% ooplasm concentrate to form a very small plaque called the GD, the accurate penetration of PVL by sperms and subsequent contact with the GD to fertilize the oocyte should be an event with a higher difficulty than in mammals. We speculated that ZP3 may be expressed at a concentration gradient to guide sperms via the PVL so as to reach the GD region. Like ZP3, ZP2 is also highly expressed in Gp cells (Table 4). This finding is in line with previous reports that ZP2 concentrates in Gp cells^58,59^. Given that ZP2 plays a role in secondary binding of sperm during fertilization in mice^57^, it is likely that ZP2 (G1p:8.62, G1d:0.22) (G5p:28.81, G5d:2.68) may play a critical role in sperm recognition and binding during fertilization in chicken as well.

**Table 4:** The FPKM value of the genes involving the extracellular matrix from G1p, G1d, G5p and G5d cells.

Gene name	G1p_FPKM	G1d_FPKM	G5p_FPKM	G5d_FPKM
***GO term: extracellular matrix-secreted proteins***				
*WNT4*	236.95	8.72	1600.32	1624.18
*IHH*	108.95	25.21	617.33	618.73
*COL14A1*	27.45	2.29	181.68	188.94
*WNT6*	16.69	0.56	28.41	7.43
*ZP2*	8.62	0.22	28.81	2.68
*SFRP1*	9.11	2.99	10.46	7.79
*AMH*	6.96	0.51	21.24	1.55
*ZP3*	82,661.90	111,496.28	14,379.31	14,816.10
*Fibronectin 1*	328.62	531.31	307.50	357.62
***GO term: extracellular matrix-transmembrane proteins/membrane anchored protein***				
*CLDN1*	18.76	0.3	18.49	1.61
*EPCAM*	21.74	0.56	29.02	7.54
*ADAMTS9*	8.42	0.78	57.84	61.55
*MMP17*	4.60	0.62	10.54	6.81
*ADAMTSL3*	3.26	0.40	2.62	0.25
*ADAMTS15*	46.89	13.57	258.56	269.10
ENSGALG00000008164	25.92	8.29	33.95	12.00
*AGRN*	13.85	4.73	21.33	10.51
Novel00781	6533.95	3297.09	11,440.58	10,462.51
*ADAMTS3*	34.71	49.91	1.95	2.72
*LTBP2*	128.32	168.10	57.87	93.83
*TNR*	2.92	7.40	0.35	0.41
*FMOD*	47.20	103.09	36.55	114.82
*TGFBI*	76.78	113.39	142.19	233.29
*CD151*	247.37	317.05	88.65	71.30

### Differential expression of signaling molecules and receptor genes in Gp and Gd cells of large preovulatory follicles

One of the particularly interesting finding in our study is that *WNT4* is predominantly and abundantly expressed in G1p cells. We found that the FPKM values of *Wnt4* (G5p:1600.32, G5d:1624.18) showed no significant difference between Gp and Gd cells in F5 follicles. In sharp contrast, in F1 follicles, *WNT4* is expressed in Gp cells nearly exclusively (G1p:236.95, G1d:8.72). As a secreted glycoprotein, *WNT4* may regulate its own expression through an autocrine route and control cell proliferation^60^. It may also regulate the proliferation of neighboring cells in a paracrine manner^61,62^. Since paracrine signaling is limited by physical distance between cells in such narrow space as the follicle, the action of WNT4 is likely restricted to neighboring Gp cells only. In Gd cells, *WNT4* expression level is much lower, hinting its limited role in these cells. Like *WNT4*, *IHH* (Indian Hedgehog Signaling Molecule) also showed a higher expression level in Gp cells (G1p:108.95, G1d:25.21). As a secreted signaling molecule, IHH was reported to regulate a variety of developmental processes including growth, patterning and morphogenesis^63^. It is likely that the proliferative effect of IHH also depends on the close contact between G1p cells, similar to that of *WNT4*.

**Figure 4:**
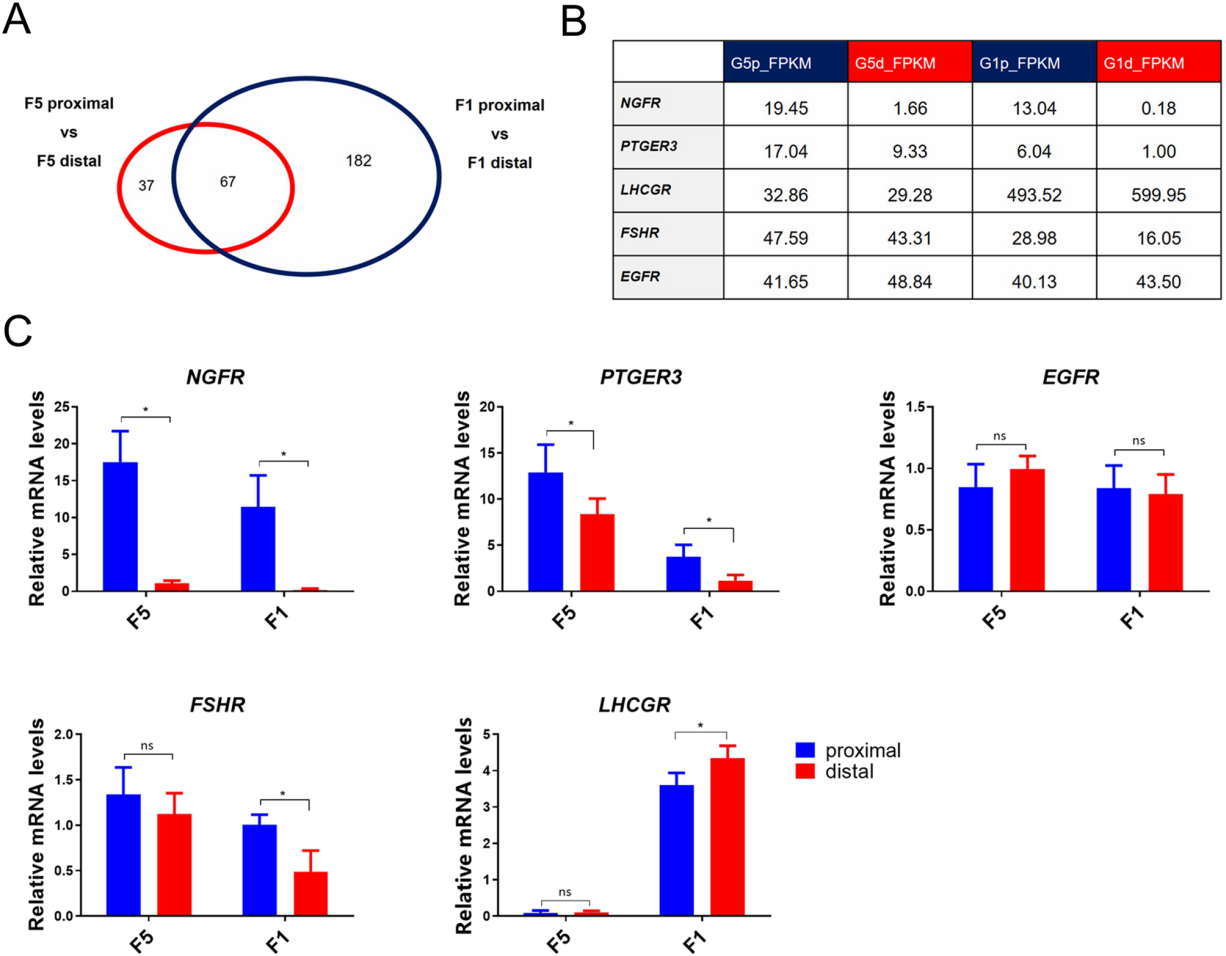
The comparison of the genes expression profiles of the Gp and Gd granulosa cells along the follicular growth. (**A**) The number of genes showed the similar expression tendency between the Gp granulosa cells and the Gd granulosa cells. (**B**) The FPKM value comparison of the genes (NGFR, PTGER3, LHCGR, FSHR and EGFR) along the follicular growth. (**C**) Quantitative real-time PCR validation of the genes expression (NGFR, PTGER3, LHCGR, FSHR and EGFR) along the follicular growth. The results were normalized based on the housekeeping gene *GAPDH*. Each data point represents mean of ten individuals (N = 10) *p < 0.05.

In the present study, the 104 differentially expressed transcripts in F5 follicles were compared with the 249 transcripts differentially expressed in F1 follicles. A total of 67 transcripts showed a clear consistent regulation tendency between the two granulosa subpopulations (Gp and Gd) in both follicles (Fig. 4A). Among the transcripts, the receptor genes *NGFR* and *PTGER3* were found to be differentially expressed in Gp cells and Gd cells. As shown in Fig. 4B, *NGFR* is highly expressed in Gp cells of F1 and F5 follicles (G5p: 19.45, G1p: 13.04), however, it is weakly expressed in Gd cells of F5 and F1 follicles (G5d: 1.66, G1d: 0.18). This finding was further confirmed by qRT-PCR analyses (Fig. 4C). *NGFR* can activate Raf kinase-MAPK/ERK signaling cascade^64,65^. Although there has not been adequate evidence supporting the hypothesis that this receptor regulates the proliferation of granulosa cells, there was a previous report suggesting that NGFR signaling plays a significant role in the shaping of neural tube in chick embryos^66^. Future study on the roles of NGFR signaling in the control of Gp proliferation and functions will be required. Similar to that of *NGFR*, the gene encoding prostaglandin E2 receptor subtype 3 (PTGER3) also displays a differential expression profile in Gp and Gd cells and its expression are much lower in F1 follicles (G1p: 6.04, G1d: 1.00) (G5p: 17.04, G5d: 9.33). It is reported that, like mammalian PTGER3, chicken PTGER3 activation can inhibit cAMP accumulation and stimulates the MAPK/ERK pathway^67^. The differential expression of PTGER3 also hints that PTGER3 signaling and its activation of the downstream MAPK/ERK signaling pathway may be involved in the control of granulosa cell proliferation.

In this study, as shown in Fig. 4B, the FPKM values of the transcript encoding *LHCGR* in F5 follicles (G5p: 32.86, G5d: 29.28) are much lower than in F1 follicles (G1p: 493.52, G1d: 599.95). *LHCGR* showed a distinct differential expression between Gp and Gd in F1 follicles. Our finding is consistent with previous studies, in which Gd cells were shown to have higher *LHR* mRNA levels and show higher responsiveness to LH treatment in term of P4 production in F1 follicles^10,68^. The high abundance of LHR is likely to coordinate the surge of LH, thus initiating the oviposition process^69^. Our findings support that the pituitary gonadotropin LH is most likely involved in the control of cell heterogeneity and functional diversity of GC through LHR signaling. Along follicular development, granulosa cells change from FSHR dominance to LHR dominance^70^. In line with this tendency, as shown in Fig. 4B, the expression of FSHR are lower at the later follicular stage in both Gp and Gd cells. Similar to the expression profile of *LHCGR*, the expression of FSHR also showed the differential pattern between the two cell subpopulations in F1 follicles (Fig. 4B), suggesting its possible role in the heterogeneity and functional diversity observed between Gp and Gd cells.

EGFR signaling has been reported to play critical roles in the control of ovarian granulosa cell proliferation, differentiation and apoptosis in chickens, mammals and teleosts^71^. In this study, we found that EGFR is abundantly expressed in both Gp and Gd cells. In addition, no statistical difference in EGFR expression levels was detected between Gp and Gd cells of F5 and F1 follicles. Our finding contrasts the report of a previous study, in which EGFR was shown to be differentially expressed in Gd and Gp cells^14^. In addition, our finding is slightly different from another report that the EGFR expression level is high in small follicles and small yellow follicles, but decreases in F2 and F1 follicles^72^*. *The F1 and F5 follicles harvested for the transcriptome analysis in our study are the large follicles belonging to the third and the final growth phase of ovarian follicles. To meet the need of the fast growth of large preovulatory follicles and keep pace with the rapid yellow yolk incorporation into these large follicles, it was suggested that Gd cells may increase its intercellular space and modulate its cell morphology^8^. The abundant consistent expression of EGFR in both Gp and Gd cells strongly supports the role of EGFR signaling in granulosa cell proliferation to cover the growing oocyte surface in large preovulatory follicles. However, the abundant expression of EGFR in Gd and Gp cells raises an interesting question regarding the source of EGFR ligands. It was reported that the GD secretes factors to regulate granulosa cell proliferation and steroidogenesis^12^. Their immunocytochemical analysis demonstrated that EGF is present in the GD. Our previous study revealed that HB-EGF, an EGFR ligand, is abundantly expressed in the oocyte and granulosa cells. In vitro studies revealed that EGF and other EGFRL (eg HB-EGF) can regulate granulosa cell proliferation, inhibit apoptosis, and decrease basal P4 production^73^. On the other hand, the addition of anti-EGF antibody abolished the ability of the GD-conditioned medium to stimulate granulosa cell proliferation^68^. All these findings, together with the report of EGFR ligands are either derived from the oocyte (or GD) or granulosa cells, support the hypothesis of the continual involvement of EGFR signaling pathway in regulating granulosa cell proliferation, differentiation, and function along follicular growth.

Taken together, in chicken preovulatory follicles, GD region occupies less than 1% of oocyte volume^7^ , but it is likely the developmental center and source of multiple signals keeping the follicles active for further development^30,74^. Gp cells, which are proximal to the GD, may keep the oocyte-granulosa cell communication active through secretion of local paracrine factors, such as oocyte-derived HBEGF, GDF9 and BMP15^68^ via gap junctions^8^. Our study revealed that genes encoding local factors such as *WNT4*,* IHH*,* NGF and AMH* are also differentially expressed, and they are likely involved in the control of Gp cell proliferation and differentiation. Gd cells which are distal to the GD is likely to have fewer communication with the oocyte (or GD), therefore, their differentiation and proliferation may rely on pituitary hormones (eg FSH/LH) and local factors derived from somatic cells other than the GD.

In a brief summary, the transcriptomes of Gp and Gd from F1 and F5 follicles were analyzed in this study. We found that genes related to cell cycle and DNA replication are preferentially expressed in Gp cells, particularly in F1 follicles, while genes associated with steroidogenesis are preferentially expressed in Gd cells. These findings clearly indicate that Gp cells (especially G1p cells) is highly proliferative with a lower steroidogenic capacity, whereas Gd cells are of a higher steroidogenic capacity and is less mitotically active. In addition, genes associated with extracellular matrix organization, paracrine/autocrine signaling, and sperm binding are differentially expressed in Gp and Gd cells. All these findings, together with the detection of the abundant/differential expression of receptors for pituitary hormones (eg LHR/FSHR) or local factors derived from the GD or somatic cells (eg EGFR, NGFR) in two granulosa cell subpopulations, provide substantial evidence that Gp and Gd cells differs significantly in their mitotic activity, sperm binding, steroidogenesis and morphology.

## Supplementary information

Supplementary information.
