# Supplementary material for: Transcriptomic analysis of granulosa cell populations proximal and distal to the germinal disc of chicken preovulatory follicles
Source: Sci Rep. 2021 Feb 25;11:4683. doi: 10.1038/s41598-021-84140-w (PMC7907084; doi:10.1038/s41598-021-84140-w)
Supplement: Supplementary file 1 — Supplementary information. [file 41598_2021_84140_MOESM1_ESM.pdf]

Transcriptomic Analysis of Granulosa Cell Populations Proximal and Distal to the Germinal Disc of Chicken Preovulatory Follicles

Guoqiang ZHU[1], Chao FANG[1], Chunheng MO, [1] Yajun WANG[1], Yan HUANG*[2], Juan LI*[1]

([1]Key Laboratory of Bio-resources and Eco-environment of Ministry of Education, College of Life Sciences, Sichuan University, Chengdu 610065, PR China; [2]The China Conservation and Research Center for the Giant Panda, Wolong, PR China)

Keywords: Chicken, ovary, follicle development, granulosa cell, transcriptome analysis

Running title: transcriptome analysis of chicken granulosa cells

** Correspondence to:*

Prof. Juan LI

Key Laboratory of Bio-resources and Eco-environment of Ministry of Education,

College of Life Sciences, Sichuan University, Chengdu, 610064, PR China

Email: lijuanscuhk@163.com

** Correspondence to:*

Prof. Yan HUANG

The China Conservation and Research Center for the Giant Panda, Wolong, PR China

Email: pandayard@hotmail.com

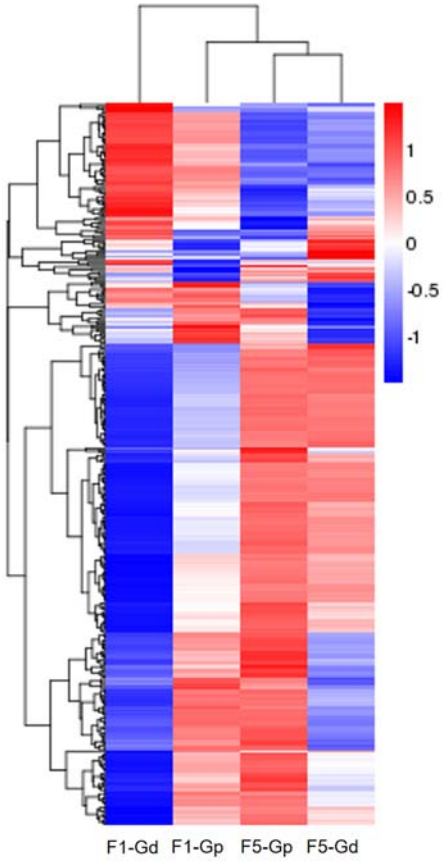

Fig S1

The heatmap displaying the gene expression profiles between chicken granulosa cells proximal (Gp) and distal(Gd) to the germinal disc in F1 and F5 follicles. The FPKM were directly from the chicken RNA-seq data (accession number: GSE118886)

Table S1: **The FPKM value of the genes showing the similar expression tendency along the follicular growth between the granulosa cells proximal and distal to the germinal disc.**

Gene Name/Gene_ID	G1p_FPKM	G1d_FPKM	G5p_FPKM	G5d_FPKM
ABCC4	12.99	1.61	3.80	0.23
ADAMTSL3	3.26	0.40	2.62	0.25
AGRN	13.85	4.73	21.33	10.51
AMH	6.96	0.51	21.24	1.55
ARHGDIG	26.95	6.72	33.34	9.07
B3GNT7	18.84	2.58	13.83	1.28
CA9	21.39	0.90	47.49	17.76
CARHSP1	30.42	7.04	36.32	11.77
CCNB2	97.71	5.80	104.50	35.44
CCNO	51.75	1.18	54.96	5.43
CDC20	43.86	11.99	79.88	49.84
CDH3	51.37	3.45	85.78	35.68
CLDN1	18.76	0.30	18.49	1.61
COLGALT2	27.04	13.67	19.96	7.83
CYP11A1	322.42	632.87	132.73	201.56
DGAT2	18.65	0.60	25.30	7.23
EMCN	3.67	0.12	10.62	0.99
ENG	7.40	0.11	11.51	0.45
ENSGALG00000007703	5.45	0.05	6.19	1.04
ENSGALG00000008164	25.92	8.29	33.95	12.00
ENSGALG00000023172	17.87	0.83	80.50	9.93
ENSGALG00000027090	9.14	15.65	46.72	17.02
EP3	6.04	1.00	17.04	9.33
EPCAM	21.74	0.57	29.02	7.54
FAM101B	12.89	0.48	16.07	1.31
FAM47E-STBD1	52.11	14.29	37.13	11.56
FAR-1	50.58	70.93	23.19	38.54
FMOD	47.20	103.09	36.55	114.82
FNDC1	38.50	50.56	22.52	32.15
GMNN	13.46	2.64	19.89	9.10
GPX3	972.06	476.05	393.48	235.54
GRAMD2	58.43	29.46	58.73	28.90
H-RAS	119.83	66.60	107.63	44.48
Id1	185.10	52.59	280.06	50.54
IRF6	15.63	0.17	17.33	2.32
LMO2	7.32	1.64	10.18	2.60

LRP1	123.04	158.40	40.08	65.38
LTBP2	128.32	168.10	57.87	93.83
MCAM	25.90	1.61	40.13	13.33
MRC2	23.80	0.28	24.21	3.01
MYCL	34.50	15.99	38.73	13.41
NBL1	45.32	2.40	51.15	5.83
NGFR	13.04	0.18	19.45	1.66
Novel00119	23.04	9.89	13.98	4.62
Novel00730	6.61	0.03	8.08	0.21
Novel00960	26.04	55.46	46.15	83.09
Novel00987	12.60	2.66	19.99	4.70
OLFM3	9.47	2.22	19.48	7.16
PIK3IP1	44.50	8.19	79.45	30.16
PLK3	19.29	1.94	18.13	2.43
PLTP	779.80	1235.24	232.23	345.53
PPP1R3B	2.56	0.03	3.29	0.24
RBPMS2	9.12	1.07	16.51	2.94
RETSAT	53.03	73.83	58.67	84.84
SGK2	10.70	0.60	37.33	14.78
SIDT1	4.28	0.03	8.52	1.64
SIKE1	33.17	10.15	35.84	15.38
SLC25A6	213.05	280.41	150.78	220.05
SLC8B1	42.05	21.02	50.31	25.81
STC1	672.13	1221.45	50.97	103.33
SVEP1	1.42	0.07	7.40	3.40
TGFBI	76.78	113.39	142.19	233.29
TMEM184A	9.66	0.45	10.50	0.41
TNIK	18.56	4.87	32.36	18.39
Wnt6	16.69	0.56	28.41	7.43
WT1	4.51	0.08	4.62	0.38
ZP2	8.62	0.22	28.81	2.68

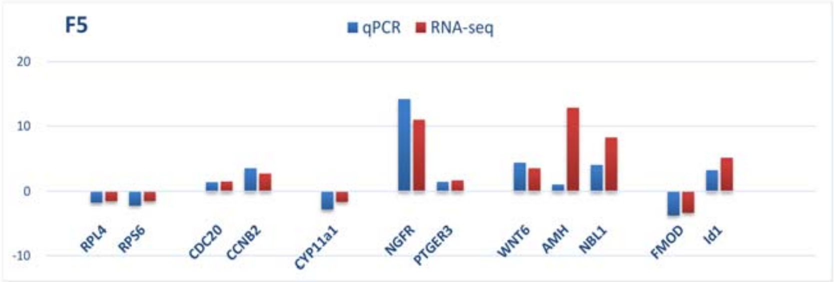

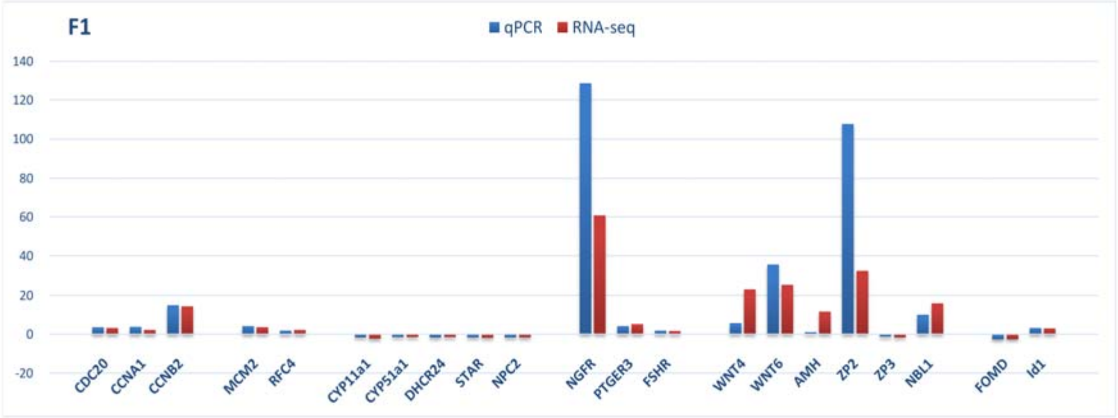

Figure S2

The comparison of differentially expressed genes validated by quantitative real-time PCR (qPCR) and RNA-seq between Gp and Gd cells of preovulatory (F5 and F1) follicles.
